# Recurrent reproductive failure and celiac genetic susceptibility, a leading role of gluten

**DOI:** 10.3389/fimmu.2024.1451552

**Published:** 2024-10-24

**Authors:** Eduardo de la Fuente-Munoz, Miguel Fernández-Arquero, Nabil Subhi-Issa, Kissy Guevara-Hoyer, Lydia Pilar Suárez, Raquel Gil Laborda, Marina Sánchez, Juliana Ochoa-Grullón, María Guzmán-Fulgencio, Ángela Villegas, María Dolores Mansilla, Noelia Pérez, Ricardo Savirón Cornudella, Teresa Gastañaga-Holguera, Marta Calvo Urrutia, Ignacio Cristóbal García, Silvia Sánchez-Ramón

**Affiliations:** ^1^ Department of Clinical Immunology, Instituto de Medicina de Laboratorio (IML) and Fundación para la Investigación Biomédica del Hospital Clínico San Carlos (IDISCC), Hospital Universitario Clínico San Carlos, Madrid, Spain; ^2^ Department of Immunology, Ophthalmology and Otorhinolaryngology (ENT), School of Medicine, Universidad Complutense, Madrid, Spain; ^3^ Cancer Immunomonitoring and Immune-mediated Diseases Research Unit, Instituto de Investigación Sanitaria San Carlos (Fundación para la Investigación Biomédica del Hospital Clínico San Carlos (IDISCC)), Department of Clinical Immunology, Hospital Universitario Clínico San Carlos, Madrid, Spain; ^4^ Department of Obstetrics and Gynecology, Hospital Universitario Clínico San Carlos, Madrid, Spain

**Keywords:** recurrent reproductive failure (RRF), non-celiac-gluten-sensitivity (NCGS), gluten, celiac disease (CD), HLA

## Abstract

**Introduction:**

The prevalence of gluten-related disorders, mainly celiac disease (CD) and non-celiac gluten sensitivity (NCGS), varies between 0.6% and 13% in the general population. There is controversial evidence regarding the association of both CD and NCGS with extra-digestive manifestations, including recurrent reproductive failure (RRF), which may have clinical implications.

**Objective:**

To study the prevalence of HLA susceptibility alleles for CD/NCGS in a cohort of female patients with RRF from a single reference center and to evaluate the effect of a gluten-free diet on reproductive success.

**Material and methods:**

A retrospective study was conducted on 173 patients with RRF, consecutively attended at the Reproductive Immunology Unit of San Carlos University Clinical Hospital in Madrid. We collected and analyzed the clinical, analytical, and immunological profiles of RRF patients who presented HLA alleles associated with CD and NCGS (HLA DQ2.2, DQ2.5, DQ8, and DQ7.5).

**Results:**

We observed a significantly higher prevalence of HLA alleles associated with CD and NCGS in our RRF cohort compared to the prevalence in the general population (69% vs. 35%–40%, p<0.0001). Only 2.3% of patients met the criteria for a CD diagnosis. In our RRF cohort, HLA-genetic susceptibility for CD/NCGS (HLA-risk group) was associated with a significantly higher rate of hypothyroidism compared to patients without these alleles (HLA-negative group) (48.7% vs. 26.92%, p=0.03). Patients with HLA-genetic susceptibility for CD/NCGS and thyroid disease had a significantly higher success rate in the subsequent pregnancy after management (55% vs. 30%, p=0.002). Two factors were found to be significant in this group: a gluten-free diet (p=0.019) and the use of levothyroxine (p=0.042).

**Conclusions:**

In our cohort of RRF patients, we observed a significantly higher prevalence of HLA susceptibility genes for CD/NCGS compared to the general population, also associated with a higher incidence of thyroid alterations. A gluten-free diet and the use of levothyroxine in cases of thyroid pathology had significant beneficial effects on pregnancy outcomes. We suggest that HLA typing for CD/NCGS and a gluten-free diet, in the presence of risk alleles, can improve pregnancy outcomes in RRF patients.

## Introduction

Celiac disease (CD) is an immune-mediated disorder associated with gluten intake, primarily manifesting with digestive symptoms, that affects 0.6% –1% of the general population. Currently, there are several methods for diagnosing and monitoring CD, including duodenal biopsy (histopathology and study of epithelial lymphogram) or peripheral blood tests (anti-transglutaminase antibodies, anti-gliadin antibodies, anti-endomysial antibodies, or HLA class II typing for identifying CD-related genetic susceptibility) (1, 2) However, despite these advancements and the growing knowledge of the disease, CD is still considered underdiagnosed, which may lead to long-term complications (3). Cumulative evidence from the last few decades suggests that the spectrum of CD represents a multisystemic disease, with multiple organs and tissues affected (such as the skin, kidneys, nervous system, or endocrine system) (1).

The cornerstone of treatment to date is to follow a strict gluten-free diet, which usually controls the different symptoms and minimizes the risk of severe complications, such as intestinal lymphoma (1, 2).

More recently, the spectrum of conditions related to CD has expanded to include other pathologies associated with wheat and/or gluten intake, such as gluten allergy and non-celiac gluten sensitivity (NCGS). Gluten allergy is an IgE-mediated type 1 hypersensitivity disease to gluten antigens. It has a well-established pathophysiological basis and specific biomarkers. However, these parameters are not as clearly defined in NCGS (1, 4, 5). NCGS is thus considered an emerging systemic (both intestinal and extraintestinal) disease in the context of wheat intake, affecting patients who do not meet the diagnosis criteria for CD or gluten allergy, often due to the absence of autoantibodies. As with CD, symptoms improve or subside when gluten is removed from the diet (4, 6). The correlation between NCGS and HLA is not well understood, although some studies show a correlation of up to 100% in these patients (4). NCGS prevalence varies between 0.6% and 13% in the general population (5).

There is compelling evidence that CD can cause reproductive alterations in men (7) and especially in women, such as sterility, intrauterine growth restriction, and miscarriages (8–12). However, very few studies have assessed the effects of NCGS and HLA-genetic susceptibility for CD/NCGS on the reproductive health of couples (13).

According to the World Health Organization, infertility and sterility affect millions of people worldwide and have a significant negative impact on both individuals and society (14, 15). There is no precise data on the prevalence of these pathologies, although it is estimated that only 30% of pregnancies reach term (16) and that between 2% and 5% of couples suffer recurrent miscarriages (17). The causes of these fertility problems can be multiple, including autoimmune alterations among others (anatomical, hormonal, infectious, genetic, and nutritional) (16). In approximately 50% of recurrent miscarriage cases, the underlying factor remains unidentified, highlighting the need for continued research into new causes, associations, and potential biomarkers.

In this study, we aimed to determine the prevalence of HLA-genetic susceptibility for CD and NCGS in a real-life cohort of patients with RRF. We also sought to better characterize this subgroup of patients and describe the potential beneficial effects of gluten withdrawal from the diet on reproductive success.

## Materials and methods

### Study design

This is a retrospective observational study, conducted at the Reproductive Immunology Unit, Clinical Immunology Department of the San Carlos Clinical University Hospital in Madrid. The data of 173 patients who were consecutively studied between February 2018 and April 2022 were analyzed. Recurrent pregnancy loss (RPL) was defined as the loss of two or more pregnancies, including non-visualized pregnancy losses, in accordance with the European Society for Human Reproduction and Embryology (ESHRE) Guidelines. Repeated implantation failure (RIF) was defined as the failure to achieve a clinical pregnancy after more than three high-quality embryo transfers or after the transfer of ≥10 embryos in multiple transfers in women under 40 years old. Fetal death (FD) was defined as a composite outcome that included women with a history of late fetal loss (between 22 weeks and 28 weeks of pregnancy) and stillbirth (after 28 weeks gestational age).

The following data were collected from the clinical histories: age; personal history with special emphasis on the diagnosis of CD; gluten consumption; digestive, neurological, dermatological, gynecological, and endocrinological diseases or symptoms; smoking habit; previous clinical diagnoses; number of miscarriages; number of *in vitro* fertilization (IVF) cycles; and anatomical/infectious alterations detected by ultrasound or hysteroscopy. Endometriomas and adenomyosis were classified as endometriosis. Endometritis was diagnosed via hysteroscopy, supported by microbiological culture and biopsy confirming the presence of CD138^+^ cells.

All patients underwent a baseline immunological study as part of the routine clinical workup for RRF, which included the detection of antinuclear antibodies, anti-thyroid antibodies (anti-thyroid peroxidase and anti-thyroglobulin antibodies), IgA anti-transglutaminase, IgG anti-deamidated gliadin and antiphospholipid antibodies (including IgM and IgG anti-cardiolipin and IgM and IgG anti-B2 glycoprotein antibodies), C3 and C4 complement levels, and relative and absolute values of NK cells in peripheral blood. HLA class II typing for identifying CD/NCGS- related genetic alleles (DQ2.2, DQ2.5, DQ8, and DQ7.5) was also performed. Additionally, other factors such as basic coagulation parameters, basal blood glucose and insulin, TSH and free T4 levels, and vitamin levels (B9, B12, and vitamin D) were collected and analyzed.

We analyzed the prevalence of alleles associated with susceptibility to CD/NCGS (HLA DQ2.2, DQ2.5, DQ8, and DQ7.5) and correlated these with analytical parameters and clinical data, such as alterations in the glucose profile, thyroid, presence of endometritis or endometriosis, gastrointestinal or neurological symptoms (specifically chronic migraines), skin alterations, polycystic ovary syndrome, presence of antiphospholipid antibodies, antinuclear antibodies, or expanded cytotoxic NK cells.

Alteration of the glycemic profile was defined as a basal glycemia higher than 100 mg/dL or a Homeostatic Model Assessment (HOMA) higher than 3. The HOMA was calculated using the formula: (glucose × insulin)/405.

Thyroid abnormalities were assessed when TSHs were above 2.5 uIU/ mL or when thyroid-specific autoantibodies were detected. Previous diagnosis of hypothyroidism was also considered (18–20).

Gestational success was defined as the birth of a live newborn at or beyond 37 weeks of pregnancy. The pregnancy success rate was determined in the next 12 months following the evaluation.

The Ethics Committee of our hospital approved the study protocol (FIS PI19/01450), and all subjects provided signed informed consent.

### Laboratory tests

Autoimmunity tests were performed as routine samples in the Clinical Immunology laboratory using specific technologies and procedures. For anti-transglutaminase, anti-deamidated gliadin, and anti-peroxidase and anti-thyroglobulin antibodies determination, an ELISA (AESKU.GROUP, Wendelsheim, Germany) technique was used. The detection of antinuclear antibodies was performed by indirect immunofluorescence (AESKU.GROUP, Wendelsheim, Germany). The antiphospholipid antibodies were detected using Luminex technology (Bio- Rad Laboratories, Hercules, CA, USA), the evaluation of the complement system by turbidimetry (The Binding Site Group Ltd., Birmingham, UK), and the determination of NK (CD3^−^CD16^+^CD56^+^) lymphocyte values by flow cytometry (Becton-Dickinson, San Jose, CA, USA). All techniques were performed and validated following the manufacturer’s instructions.

BD MultiTEST™ CD3 fluorescein isothiocyanate (FITC)/CD16^+^CD56 phycoerythrin (PE)/CD45 peridinin chlorophyll protein (PerCP)/CD19 allophycocyanin (APC) was used to study the NK cells. First, NK cells were gated by singlets and CD45^+^ and side scatter appropriate for lymphocytes. Then were gated by CD3 ^−^ and last for CD16^+^ and CD56^+^ and CD19 ^−^. The absolute values of NK cells were calculated through the relative value obtained by flow cytometry and the absolute value of total lymphocytes in the blood count. The cutoff point used to consider pbNK cells expanded was 13%.

In order to extract the DNA from fresh peripheral blood leukocytes, we used MagNA Pure Compact Nucleic Acid Isolation Kit (Roche®, Darmstadt, Germany) following the manufacturer’s procedures. All samples were genotyped for HLA-DRB1, HLA-DQA1, and HLA-DQB1 by polymerase chain reaction–sequence-specific oligonucleotide probe (PCR-SSOP) (Thermo Fisher, Waltham, USA), where PCR products were hybridized onto oligonucleotide probes attached to microspheres and labeled with streptavidin-conjugated phycoerythrin. These beads were analyzed with the Luminex® 100/200 TM System (Luminex Corp., Austin, TX, USA), which is based on flow cytometry and uses the principles of xMAP® Technology, as previously described (21).

The allelic results obtained were analyzed by an immunology specialist. The haplotypes were classified according to the following distribution: DQ2.5, DQA1 0501/0505 DQB1 0201/0202; DQ2.2, DQA 0201 DQB1 0201/0202; DQ8, DQA1 0301/0302 DQB1 0302; and DQ7.5, DQA1 0501/0505 DQB1 0301.

### Statistical analysis

Descriptive data are presented as median ± standard deviation (SD). Statistical Product and Service Solutions (SPSS) software version 20 (Chicago, IL, USA) was used for descriptive and statistical data analysis. Comparisons between groups were made using the chi-square test (χ^2^); median comparison were made using Student “t” test; p ≤ 0.05 was considered statistically significant.

## Results

### Epidemiological and obstetric features

Of the 173 patients with recurrent reproductive failure analyzed, 112 had recurrent miscarriages (RMs), 51 had recurrent implantation failure (RIF), and five patients were referred preventively due to underlying immune pathology with risk of miscarriages or obstetric complications and five due to a history of previous fetal death. The mean age of the patients was 38.02 years.

Noteworthy, 121 patients out of 173 (69.94%) presented HLA-genetic susceptibility for CD/NCGS (HLA-DQ2.2, DQ2.5, DQ8, and/or DQ7.5), namely, HLA-risk group, while 52 patients (30.06%) did not present any of these alleles, HLA-negative group. No significant differences in age were found between both groups. Within the HLA-risk group, the distribution of the different haplotypes was as follows: DQ2.5 of 17.35%, DQ2.2 of 33.05%, DQ8 of 19.00%, and DQ7.5 of 12.39%. Additionally, 18.18% of the cohort had the presence of two different risk alleles.

In the HLA-risk group, 63.6% presented RM, 31.4% RIF, 2.48% history of FD, and 2.48% were referred due to underlying immune disorders associated with risk of RM. With respect to CD prevalence, 4 out of the 173 (2.3%) patients had been previously diagnosed with CD: three of them presented RM, and one previous FD. No statistical differences in obstetric morbidity were found compared to the HLA-negative group (**Table 1**).

**Table 1:** Epidemiological, clinical data and obstetric features of the HLA-susceptibility group and HLA-negative group patients.

Variable	HLA-risk group N = 121	HLA-negative group N = 52	*p*-value
**Age (years)**	38.14 ± 3.55	37.73 ± 4.35	0.25
Obstetric features			
** RM**	77 (63.63%)	35 (67.30%)	0.6
** RIF**	38 (31.4%)	13 (25.00%)	0.39
** Fetal death**	3 (3.84%)	2 (3.84%)	0.6
** Previous miscarriages**	1.86 ± 1.72	1.76 ± 1.35	0.71
** Previous embryo transfer**	1.72 ± 2.14	1.30 ± 1.75	0.21
** Dysmenorrhea**	73 (60.3%)	31 (59.6%)	0.92
** Endometritis**	14 (11.57%)	3 (5.76%)	0.23
** Endometriosis**	12 (9.91%)	5 (9.8%)	0.95
Clinical and analytical features			
** Hypothyroidism**	59 (48.76%)	17 (26.92%)	0.03*
** Expanded cytotoxic pbNK**	43 (35%)	19 (36%)	0.76
** Positive ANA**	19 (15.7%)	12 (23%)	0.24
** Positive antiphospholipid antibodies**	23 (19%)	11 (21%)	0.74
** Positive anti-thyroid antibodies**	19 (15.7%)	5 (9.6%)	0.28
** Chronic migraine**	38 (31.40%)	18 (35%)	0.59

Values are expressed as median (SD) and absolute count (percentage). χ^2^ test was performed. *p ≤ 0.05 was considered statistically significant. RMs, recurrent miscarriages; RIF, recurrent implantation failure; ANA, antinuclear antibodies ; pbNK, peripheral blood natural killer.

Bolded values indicate statistical significance with p < 0.05.

Regarding clinical manifestations, the HLA-risk group presented significantly higher thyroid abnormalities compared to the HLA-negative group (48.76% vs. 26.92%, p=0.0368). However, no statistically significant differences were found between the HLA-risk group and the HLA-negative group for other parameters, such as glucose alterations, endometritis, endometriosis, dysmenorrhea, polycystic ovary syndrome, gastrointestinal symptoms, chronic migraines, presence of antiphospholipid antibodies, antinuclear antibodies, nor expanded percentages of cytotoxic NK lymphocytes (**Table 1**).

Only two patients were positive for anti-transglutaminase IgA antibodies and one for anti-deamidated gliadin IgG antibodies, all of them in the HLA-risk group.

In the HLA-risk group, successful pregnancy was achieved in 67 patients (55.37%). Two statistically significant differences were observed: first, gluten intake emerged as a key factor, with the success group having a higher percentage of patients on a gluten-free diet (p=0.01). The exclusion of gluten intake in HLA-risk patients was associated with an increased likelihood of gestational success, yielding an odds ratio (OR) of 2.791 (IC, 1.166–6.679; p=0.02). Second, the use for levothyroxine also showed a significant impact. Of the 56 (46.2%) patients with TSH levels above 2.5 who were treated with levothyroxine, 37 achieved a successful pregnancy (p=0.042), as detailed in **Supplementary Table S1**. However, no statistically significant differences were found in levothyroxine use when comparing the HLA-risk group with the non-HLA risk group (46.2% vs. 36.53%, p=0.17). Of these patients, 38 underwent IVF due to a prior diagnosis of RIF. For IVF patients, a gluten-free diet was recommended at least 1 month before the embryo transfer, whereas, in other cases, the diet was introduced immediately following their evaluation.

Within the HLA-risk group, 33 women withdrew the intake of gluten, of which 24 (72.72%) achieved a successful pregnancy. A total of 88 women did not make any change in their diet, and 43 (48.86%) of them achieved a successful pregnancy (p=0.019) (**Table 2**).

**Table 2:** Gestational success and diet of HLA-risk group.

Pregnancy outcome	Gluten- free diet	Normal diet
**No. (n=121)**	33	88
**Gestational success**	24 (72.72%)*	43 (48.86%)
**Unsuccessful**	9 (27.27%)	45 (51.13%)

Chi-square test value (χ^2^)=5.531, *p=0.019 (p ≤ 0.05 was considered statistically significant).

Bolded values indicate statistical significance with p < 0.05.

No statistical differences were observed regarding the use of levothyroxine in the HLA-risk group with gluten-free diet or normal diet (51% vs. 44%, p=0.47).

## Discussion

In this real-life retrospective observational study, the first notable finding in our cohort is the high prevalence of HLA susceptibility alleles for CD/NCGS, 69.94%, almost doubling the 35%–40% prevalence reported in our population (22–25). Among patients in the HLA-risk group, a significantly higher incidence of thyroid pathology was observed. Additionally, it is noteworthy that both gluten withdrawal and levothyroxine replacement therapy, when necessary, were associated with a significantly higher rate of pregnancy success compared to patients who did not receive such treatment.

Thyroid disorders have also been described as a risk factor for pregnancy loss, and levothyroxine therapy is the standard treatment in these cases. Indeed, patients who received this drug in the HLA-risk group showed a higher pregnancy success rate. Few previous studies have explored the use of a gluten-free diet to treat patients with Hashimoto’s thyroiditis with good results in hormone levels (26, 27). HLA-DQ2 and HLA-DQ8 present gluten peptides to CD4 ^+^ T cells in the intestinal lamina propria, inducing immune activation and TH1 cell differentiation, which, in turn, drives an inflammatory process through the production of the inflammatory cytokines interferon (IFN)-γ and interleukin (IL)-21 (28). As previously mentioned, the line between non-celiac and seronegative gluten sensitivity is difficult to distinguish, especially since the pathophysiology is not yet fully understood. The data, *a priori*, suggest that the immune response is based more on the innate component, through toll-like receptors (TLR1 and TLR2 ) but presents an adaptive immunity factor through cytokines such as IFN-y and IL-15 (1, 5). It is important to highlight the role of NK cells during implantation of the fetus and placentation, as they play a fundamental part in the uterine tissue remodeling processes (29–31). The immunological alterations described preferentially affect this cell lineage (32–34). Although IL-15 is essential for the development of NK cells, continuous presence can lead to a functional defect in these cells due to exhaustion caused by a metabolic defect (35, 36).

This inflammatory response is not localized as in CD; however, since it is less intense, the effects may not be as visible or detectable. In fact, NCGS has also been associated with systemic autoimmunity symptoms such as psoriasis, thyroid disease, polyarthritis, antiphospholipid syndrome, rheumatoid arthritis, systemic sclerosis, and mixed connective tissue disease. Additionally, there have been cases where these diseases have improved significantly with the removal of gluten from the diet, even though they were refractory to immunosuppressive therapy (37).

There were no statistically significant differences in clinical manifestations between the two groups nor was the HLA-risk group associated with other typical alterations of CD such as endometritis, altered blood glucose, or the presence of ANAs or antiphospholipid antibodies (38–41).

CD and gluten-related conditions are prevalent in our environment and are on the rise due to various factors (1–3). Several studies have expressed concern that it may be an underdiagnosed disease (2, 3) and the importance of increasing awareness of atypical or extraintestinal manifestations, or even silent disease, which may progress to severe complications such as intestinal lymphoma.

Gluten-related disorders in human reproduction are still under investigation. Most studies have been conducted on patients with a clear diagnosis of CD. Among the observed alterations, amenorrhea, early menopause, recurrent miscarriages, lower pregnancy rate, placental dysfunction, low birth weight, intrauterine growth retardation, and increased risk of cesarean section have been reported (8–12).

More studies are necessary to determine the underlying pathophysiological mechanisms, which may be related to dysbiosis, inflammation, or even malabsorption, and the subsequent deficiency in micronutrients or a combination of these factors. Although the exact pathophysiological mechanisms by which these alterations occur are not completely known; in celiac patients, there is an improvement, as with the rest of the symptoms, with the withdrawal of gluten (11).

In recent years, the term polyautoimmunity has gained importance, which is defined as the presence of more than one well-characterized autoimmune disease in the same patient (42). As with other autoimmune diseases, where associations between multiple diseases with overlapping symptoms or laboratory abnormalities are observed, similar phenomena can be observed in celiac disease (1). A higher prevalence of autoimmune thyroid disease or type 1 diabetes mellitus in these patients is well documented and has been attributed to the genetic link between HLA-DQ2 and/or DQ8 and DR3 and DR4 (26). Additionally, the prevalence of autoimmune diseases in first-degree relatives is also increased, such as autoimmune thyroiditis or type 1 diabetes, as previously mentioned but also inflammatory bowel disease, Sjögren’s syndrome, lupus, Addison’s disease, autoimmune hepatitis, rheumatoid arthritis, primary biliary cirrhosis, and psoriasis among others (43).

CD has also been associated with the presence of antinuclear and antiphospholipid antibodies, known immunological factors of RRF (13, 41). This background of autoimmunity and inflammation has been described as an additional risk factor during pregnancy and the pursuit of pregnancy (44).

To date, in addition to classic CD, different forms of the spectrum have been described, such as potential CD (positive autoantibodies without mucosal lesion), silent CD (positive autoantibodies, presence of mucosal lesion but no symptoms), seronegative CD (absence of autoantibodies and mucosal lesion but with symptoms), wheat allergy (IgE-mediated pathology), and NCGS (1–3). However, some authors suggest greater complexity, suggesting that these diseases may represent expressions of a biological continuum (41).

The European guidelines for the study of CD include reproductive disorders as one of the extraintestinal symptoms of the disease. Furthermore, HLA class II typing is recommended when another immunological disease coexists (1). However, the current ESHRE guidelines do not recommend serology screening for this disease unless digestive symptoms are present, and they do not currently include HLA class II typing as part of the evaluation (45). The data obtained suggest that, in patients with suspected immunological alterations, HLA typing can provide clinically relevant information about the underlying cause and guide therapeutic measures, such as a gluten-free diet, the effectiveness of which was demonstrated in this study.

## Conclusions

Immunological alterations derived from gluten intake affect a significant percentage of the population with genetic susceptibility. Patients seeking pregnancy, especially those with RRF, are of particular interest. Our study shows a significant association between the presence of class II susceptibility alleles for CD/NCGS and RRF, and hypothyroidism. A gluten-free diet has been shown to be an effective and safe therapeutic alternative for these patients.

## Data Availability

The datasets presented in this study can be found in online repositories. The names of the repository/repositories and accession number(s) can be found in the article/**Supplementary Material**.

## Supplementary material

Supplementary Table 1 Gestational success and levothyroxine of HLA-risk Group. Chi-square test value (X2)=4.1281, *P=0.042 (p ≤ 0.05 was considered a statistically significant value).

## References

[1] Al-Toma A Volta U Auricchio R Castillejo G Sanders DS Cellier C. European Society for the Study of Coeliac Disease (ESsCD) guideline for coeliac disease and other gluten-related disorders. United Eur Gastroenterol J. (2019) 7:583–613. doi: 10.1177/2050640619844125, PMID: 31210940 PMC6545713

[2] Raiteri A Granito A Giamperoli A Catenaro T Negrini G Tovoli F. Current guidelines for the management of celiac disease: A systematic review with comparative analysis. World J Gastroenterol. (2022) 28:154–75. doi: 10.3748/wjg.v28.i1.154, PMID: 35125825 PMC8793016

[3] Choung RS Larson SA Khaleghi S Rubio-Tapia A Ovsyannikova IG King KS. Prevalence and morbidity of undiagnosed celiac disease from a community-based study. Gastroenterology. (2017) 152:830–839.e5. doi: 10.1053/j.gastro.2016.11.043, PMID: 27916669 PMC5337129

[4] Molina-Infante J Santolaria S Montoro M Esteve M Fernández-Bañares F. Sensibilidad al gluten no celiaca: una revisión crítica de la evidencia actual [Non-celiac gluten sensitivity: a critical review of current evidence. Gastroenterol Hepatol. (2014) 37:362–71. doi: 10.1016/j.gastrohep.2014.01.005, PMID: 24667093

[5] Elli L Branchi F Tomba C Villalta D Norsa L Ferretti F. Diagnosis of gluten related disorders: Celiac disease, wheat allergy and non-celiac gluten sensitivity. World J Gastroenterol. (2015) 21:7110–9. doi: 10.3748/wjg.v21.i23.7110, PMID: 26109797 PMC4476872

[6] Roszkowska A Pawlicka M Mroczek A Bałabuszek K Nieradko-Iwanicka B. Non-celiac gluten sensitivity: A review. Medicina. (2019) 55:222. doi: 10.3390/medicina55060222, PMID: 31142014 PMC6630947

[7] Farthing MJ Edwards CR Rees LH Dawson AM. Male gonadal function in coeliac disease: 1. Sexual dysfunction, infertility, and semen quality. Gut. (1982) 23:608–14. doi: 10.1136/gut.23.7.608, PMID: 7200931 PMC1419778

[8] Freeman HJ. Reproductive changes associated with celiac disease. World J Gastroenterol. (2010) 16:5810–4. doi: 10.3748/wjg.v16.i46.5810, PMID: 21155001 PMC3001971

[9] Singh P Arora S Lal S Strand TA Makharia GK. Celiac disease in women with infertility: A meta-analysis. J Clin Gastroenterol. (2016) 50:33–9. doi: 10.1097/MCG.0000000000000285, PMID: 25564410

[10] Casella G Orfanotti G Giacomantonio L Bella CD Crisafulli V Villanacci V. Celiac disease and obstetrical-gynecological contribution. Gastroenterol Hepatol Bed Bench. (2016) 9:241–9., PMID: 27895849 PMC5118848

[11] Grode L Bech BH Plana-Ripoll O Bliddal M Agerholm IE Humaidan P. Reproductive life in women with celiac disease; a nationwide, population-based matched cohort study. Hum Reprod. (2018) 33:1538–47. doi: 10.1093/humrep/dey214, PMID: 29912336

[12] Saccone G Berghella V Sarno L Maruotti GM Cetin I Greco L. Celiac disease and obstetric complications: a systematic review and metaanalysis. Am J Obstet Gynecol. (2016) 214:225–34. doi: 10.1016/j.ajog.2015.09.080, PMID: 26432464

[13] Bold J Rostami K. Non-coeliac gluten sensitivity and reproductive disorders. Gastroenterol Hepatol Bed Bench. (2015) 8:294–7., PMID: 26468350 PMC4600520

[14] Mascarenhas MN Flaxman SR Boerma T Vanderpoel S Stevens GA. National, regional, and global trends in infertility prevalence since 1990: a systematic analysis of 277 health surveys. PloS Med. (2012) 9:e1001356. doi: 10.1371/journal.pmed.1001356, PMID: 23271957 PMC3525527

[15] Boivin J Bunting L Collins JA Nygren KG. International estimates of infertility prevalence and treatment-seeking: potential need and demand for infertility medical care. Hum Reprod (Oxford England). (2007) 22:1506–12. doi: 10.1093/humrep/dem046, PMID: 17376819

[16] Ford HB Schust DJ. Recurrent pregnancy loss: etiology, diagnosis, and therapy. Rev Obstet Gynecol. (2009) 2:76–83., PMID: 19609401 PMC2709325

[17] El Hachem H Crepaux V May-Panloup P Descamps P Legendre G Bouet PE. Recurrent pregnancy loss: current perspectives. Int J Womens Health. (2017) 9:331–45. doi: 10.2147/IJWH.S100817, PMID: 28553146 PMC5440030

[18] Stagnaro-Green A Abalovich M Alexander E Azizi F Mestman J Negro R. American Thyroid Association Taskforce on Thyroid Disease During Pregnancy and Postpartum. Guidelines of the American Thyroid Association for the diagnosis and management of thyroid disease during pregnancy and postpartum. Thyroid. (2011) 21:1081–125. doi: 10.1089/thy.2011.0087, PMID: 21787128 PMC3472679

[19] Lazarus J Brown RS Daumerie C Hubalewska-Dydejczyk A Negro R Vaidya B. 2014 European thyroid association guidelines for the management of subclinical hypothyroidism in pregnancy and in children. Eur Thyroid J. (2014) 3:76–94. doi: 10.1159/000362597, PMID: 25114871 PMC4109520

[20] Taylor PN Minassian C Rehman A Iqbal A Draman MS Hamilton W. and risk of miscarriage in women on long-term levothyroxine: a community-based study. J Clin Endocrinol Metab. (2014) 99:3895–902. doi: 10.1210/jc.2014-1954, PMID: 25057882

[21] Fernandez-Arquero M Arroyo R Rubio A Martin C Vigil P Conejero L. Primary association of a TNF gene polymorphism with susceptibility to multiple sclerosis. Neurology. (1999) 53:1361–3. doi: 10.1212/wnl.53.6.1361, PMID: 10522904

[22] Núñez C Garrote J-A. Recommendations to report and interpret HLA genetic findings in coeliac disease. Rev Española Enfermedades Digestivas. (2018) 110:458–61. doi: 10.17235/reed.2018.5269/2017, PMID: 29722267

[23] Siddiqui K Uqaili AA Rafiq M Bhutto MA. Human leukocyte antigen (HLA)-DQ2 and -DQ8 haplotypes in celiac, celiac with type 1 diabetic, and celiac suspected pediatric cases. Medicine. (2021) 100:e24954. doi: 10.1097/MD.0000000000024954, PMID: 33725967 PMC7982179

[24] de Entresotos Villazán LD García Calatayud S. Estudio de la Enfermedad Celíaca en la Población Pediátrica de cantabria y sus familiares de primer grado. Gastroenterol y Hepatol. (2008) 31:53–8. doi: 10.1157/13116070, PMID: 18279642

[25] Sollid LM Lie BA. Celiac disease genetics: current concepts and practical applications. Clin Gastroenterol Hepatol. (2005) 3:843–51. doi: 10.1016/s1542-3565(05)00532-x, PMID: 16234020

[26] Krysiak R Szkróbka W Okopień B. The effect of gluten-free diet on thyroid autoimmunity in drug-naïve women with Hashimoto's thyroiditis: A pilot study. Exp Clin Endocrinol Diab. (2019) 127:417–22. doi: 10.1055/a-0653-7108, PMID: 30060266

[27] Ventura A Neri E Ughi C Leopaldi A Città A Not T. Gluten-dependent diabetes-related and thyroid-related autoantibodies in patients with celiac disease. J Pediatr. (2000) 137:263–5. doi: 10.1067/mpd.2000.107160, PMID: 10931424

[28] Voisine J Abadie V. Interplay between gluten, HLA, innate and adaptive immunity orchestrates the development of coeliac disease. Front Immunol. (2021) 12:674313. doi: 10.3389/fimmu.2021.674313, PMID: 34149709 PMC8206552

[29] Sharma S. Natural killer cells and regulatory T cells in early pregnancy loss. Int J Dev Biol. (2014) 58:219–29. doi: 10.1387/ijdb.140109ss, PMID: 25023688 PMC4306453

[30] Zhang X Wei H. Role of decidual natural killer cells in human pregnancy and related pregnancy complications. Front Immunol. (2021) 12:728291. doi: 10.3389/fimmu.2021.728291, PMID: 34512661 PMC8426434

[31] Shmeleva EV Colucci F. Maternal natural killer cells at the intersection between reproduction and mucosal immunity. Mucosal Immunol. (2021) 14:991–1005. doi: 10.1038/s41385-020-00374-3, PMID: 33903735 PMC8071844

[32] Martinez J Huang X Yang Y. Direct TLR2 signaling is critical for NK cell activation and function in response to vaccinia viral infection. PloS Pathog. (2010) 6:e1000811. doi: 10.1371/journal.ppat.1000811, PMID: 20300608 PMC2837413

[33] Mah AY Cooper MA. Metabolic regulation of natural killer cell IFN-γ Production. Crit Rev Immunol. (2016) 36:131–47. doi: 10.1615/CritRevImmunol.2016017387, PMID: 27910764 PMC5335907

[34] Carson WE Giri JG Lindemann MJ Linett ML Ahdieh M Paxton R. Interleukin (IL) 15 is a novel cytokine that activates human natural killer cells via components of the IL-2 receptor. J Exp Med. (1994) 180:1395–403. doi: 10.1084/jem.180.4.1395, PMID: 7523571 PMC2191697

[35] Wang X Zhao X-Y. Transcription factors associated with IL-15 cytokine signaling during NK cell development. Front Immunol. (2021) 12:610789. doi: 10.3389/fimmu.2021.610789, PMID: 33815365 PMC8013977

[36] Felices M Lenvik AJ McElmurry R Chu S Hinderlie P Bendzick L. Continuous treatment with IL-15 exhausts human NK cells via a metabolic defect. JCI Insight. (2018) 3:e96219. doi: 10.1172/jci.insight.96219, PMID: 29415897 PMC5821201

[37] Lerner A Freire de Carvalho J Kotrova A Shoenfeld Y. Gluten-free diet can ameliorate the symptoms of non-celiac autoimmune diseases. Nutr Rev. (2022) 80:525–43. doi: 10.1093/nutrit/nuab039, PMID: 34338776

[38] Isasi C Tejerina E Morán LM. Non-celiac gluten sensitivity and rheumatic diseases. Reumatol Clin. (2016) 12:4–10. doi: 10.1016/j.reuma.2015.03.001, PMID: 25956352

[39] Yu XB Uhde M Green PH Alaedini A. Autoantibodies in the extraintestinal manifestations of celiac disease. Nutrients. (2018) 10:1123. doi: 10.3390/nu10081123, PMID: 30127251 PMC6115844

[40] Laine O Pitkänen K Lindfors K Huhtala H Niemelä O Collin P. Elevated serum antiphospholipid antibodies in adults with celiac disease. Dig Liver Dis. (2018) 50:457–61. doi: 10.1016/j.dld.2017.11.018, PMID: 29258815

[41] Yang Y Krishna K Deshpande P Ranganathan V Jayaraman V Wang T. High frequency of extractable nuclear autoantibodies in wheat-related disorders. biomark Insights. (2018) 13:1177271918782893. doi: 10.1177/1177271918782893, PMID: 29977112 PMC6024268

[42] Sarmiento-Monroy JC Gómez-Puerta JA. Poliautoinmunidad en síndrome de Sjögren. Rev Colombiana Reumatol. (2020) 27:58–66. doi: 10.1016/j.rcreu.2020.07.003

[43] Kahaly GJ Frommer L Schuppan D. Celiac disease and endocrine autoimmunity - the genetic link. Autoimmun Rev. (2018) 17:1169–75. doi: 10.1016/j.autrev.2018.05.013, PMID: 30316996

[44] Carp HJ Selmi C Shoenfeld Y. The autoimmune bases of infertility and pregnancy loss. J Autoimmun. (2012) 38:J266–74. doi: 10.1016/j.jaut.2011.11.016, PMID: 22284905

[45] Recurrent pregnancy loss (2017). Available online at: https://www.eshre.eu/Guidelines-and-Legal/Guidelines/Recurrent-pregnancy-loss (Accessed March 01, 2024).

