# Correction: Recurrent reproductive failure and celiac genetic susceptibility, a leading role of gluten

**DOI:** 10.3389/fimmu.2025.1701918

**Published:** 2025-09-23

**Authors:** Eduardo de la Fuente-Munoz, Miguel Fernández-Arquero, Nabil Subhi-Issa, Kissy Guevara-Hoyer, Lydia Pilar Suárez, Raquel Gil Laborda, Marina Sánchez, Juliana Ochoa-Grullón, María Guzmán-Fulgencio, Ángela Villegas, María Dolores Mansilla, Noelia Pérez, Ricardo Savirón Cornudella, Teresa Gastañaga-Holguera, Marta Calvo Urrutia, Ignacio Cristóbal García, Silvia Sánchez-Ramón

**Affiliations:** ^1^ Department of Clinical Immunology, Instituto de Medicina de Laboratorio (IML) and Fundación para la Investigación Biomédica del Hospital Clínico San Carlos (IDISCC), Hospital Universitario Clínico San Carlos, Madrid, Spain; ^2^ Department of Immunology, Ophthalmology and Otorhinolaryngology (ENT), School of Medicine, Universidad Complutense, Madrid, Spain; ^3^ Cancer Immunomonitoring and Immune-mediated Diseases Research Unit, Instituto de Investigación Sanitaria San Carlos (Fundación para la Investigación Biomédica del Hospital Clínico San Carlos (IDISCC)), Department of Clinical Immunology, Hospital Universitario Clínico San Carlos, Madrid, Spain; ^4^ Department of Obstetrics and Gynecology, Hospital Universitario Clínico San Carlos, Madrid, Spain

**Keywords:** recurrent reproductive failure (RRF), non-celiac-gluten-sensitivity (NCGS), gluten, celiac disease (CD), HLA

Author “Nabil Subhi-Issa” was erroneously spelled as “Nabil Subbhi-Issa”

The original version of this article has been updated.

