# Supplementary material for: Recurrent reproductive failure and celiac genetic susceptibility, a leading role of gluten
Source: Front Immunol. 2024 Oct 24;15:1451552. doi: 10.3389/fimmu.2024.1451552 (PMC11540631; doi:10.3389/fimmu.2024.1451552)
Supplement: Supplementary Table 1 — Gestational success and levothyroxine of HLA-risk Group. Chi-square test value (X2)=4.1281, *P=0.042 (p ≤ 0.05 was considered a statistically significant value). [file Table1.docx]

**Supplementary Table 1**

Gestational success and levothyroxine of HLA-risk Group

|  | **Levothyroxine** | **Without Levothyroxine** |
| --- | --- | --- |
| **No. (n=121)** | 56 | 65 |
| **Gestational success** | 37 (30.58%)* | 31 (25.62%) |
| **Unsuccessful** | 19 (15.70%) | 34 (28.10%) |

Chi-square test value (X2)=4.1281, * P=0.042 (p≤0.05 was considered a statistically significant value).
